# Quantitative Ethnobotany of Multiple‐Use Species and Management of the Yangambi Biosphere Reserve in the Democratic Republic of the Congo

**DOI:** 10.1002/ece3.71110

**Published:** 2025-03-09

**Authors:** Daddy D. Kipute, Alain L. Katayi, Nestor K. Luambua, Jean‐Marie Kahindo, Salomon Mampeta, Ursil Lelo, Daou Véronique Joiris, Jean‐Pierre Mate

**Affiliations:** ^1^ Department of General Agronomy, Faculty of Renewable Natural Resources Management Université de Kisangani Kisangani Democratic Republic of the Congo; ^2^ Department of Crop Sciences, Faculty of Agronomy Université Officielle de Mbujimayi Mbujimayi Democratic Republic of the Congo; ^3^ Department of Agricultural Economics, Faculty of Agronomy Université Officielle de Mbujimayi Mbujimayi Democratic Republic of the Congo; ^4^ Isotope Bioscience Laboratory (ISOFYS), Department of Green Chemistry, Faculty of Bioscience Engineering Ghent University Gent Belgium; ^5^ Departement of Animals Sciences, Faculty of Agronomy Université Officielle de Mbujimayi Mbujimayi Democratic Republic of the Congo; ^6^ Wood Biology Laboratory of Yangambi Yangambi Democratic Republic of the Congo; ^7^ Department of Botany, Faculty of Sciences Université de Kisangani Kisangani Democratic Republic of the Congo; ^8^ Department of Sociology Université de Kisangani Kisangani Democratic Republic of the Congo; ^9^ Department of Economic and Social law, Faculty of law Université de Kisangani Kisangani Democratic Republic of the Congo; ^10^ Centre d'anthropologie Culturelle Université Libre de Bruxelles, Institut de Sociologie Bruxelles Belgium; ^11^ Ecole Post‐régionale d'Aménagement et de Gestion intégrés Des Forêts et Des Territoires Tropicaux (ERAIFT) Université de Kinshasa Kinshasa Democratic Republic of the Congo

**Keywords:** biodiversity conservation, ethnobotanical survey, plant use value, quantitative ethnobotany, Yangambi Biosphere Reserve

## Abstract

The Yangambi Biosphere Reserve (YBR) in the Democratic Republic of Congo (DRC) faces significant challenges regarding the livelihoods of local communities and biodiversity conservation. The lack of scientific information on the spatial distribution of useful woody species hinders sustainable forest resource management and is a development constraint. This study was conducted in the villages of Yaselia, Lilanda, and Bagbanye on the outskirts of the protected area and aimed to identify the most useful woody species, analyze their socio‐cultural value, assess their uses based on local community involvement, and evaluate their abundance beyond village forests to contribute to reforestation and conservation policies. An ethnobotanical survey of 105 households and a forest inventory of 9 ha were conducted to assess the abundance of 29 useful woody species. The results revealed that species such as *Entandrophragma cylindricum* (Sprague) Sprague and Hoyle*, Petersianthus macrocarpus* (P.Beauv.) Liben, *Ricinodendron heudelotii* (Baill.) Pierre ex Heckel, *Scorodophloeus zenkeri* Harms, *Pentaclethra macrophylla* Benth., *Uapaca guineensis* Müll.Arg., *Blighia welwitschii* (Hiern) Radlk., *Chrysophyllum lacourtianum* De Wild., *Dacryodes edulis* (G.Don) H.J.Lam, *and Gilbertiodendron dewevrei* (De Wild.) J.Léonard have high use and cultural value for local communities. The forest inventory showed that primary forests are better represented in terms of abundance and biomass of species with high use and cultural value, while fallow lands are less diverse and dominated by small‐diameter trees. However, most of the useful species identified with high use and cultural value have low density/biomass or are absent in most of the plots in secondary forests and fallow land. These results underscore the urgent need to implement sustainable management strategies that include these species through traditional agroforestry projects. Such initiatives would enhance resource valorization, support local livelihoods, and reduce pressure on the YBR, contributing to the preservation of this biodiversity sanctuary and the promotion of sustainable forest management in the region.

## Graphical abstract

Woody species with high use value are identified, and their abundance in village forests is assessed through the forest inventory. Species with high use and socio‐cultural value are in low density in village forests around the Yangambi Biosphere Reserve (YBR). Scarcity of species with high use and socio‐cultural value encourages communities to foray into the protected area of the YBR to harvest the products of these resources. Use of species with high use value in agroforestry projects around the YBR will help reduce pressure on natural resources in this protected area.

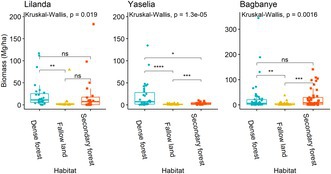

## 1 Introduction

The Yangambi Biosphere Reserve (YBR) is recognized as a famous hotspot of biodiversity, especially forest resources (Sibret et al. 2022; Luambua et al. 2021). Nevertheless, the rural populations continuously consider this protected area as a reservoir of their livelihood due to the socio‐economic interest of these woody resources for their fundamental needs (Badjaré et al. 2018). Furthermore, woody species have social, symbolic, economic, strategic, ecological, and cultural value (Katayi et al. 2023; Gautier 1994). Indeed, these resources provide non‐timber forest products (NTFP) or timber products for subsistence needs and market sales. They also contribute to poverty reduction, food security, pharmacopeia, housing construction, and income security (Sonwa et al. 2007).

Despite the crucial role of woody plants in resolving the socio‐economic needs of riparian areas and their ecological importance in mitigating the effects of climate change the rate of deforestation and degradation of tropical forests in the world, especially in the Congo basin, is still alarming (Aquilas et al. 2022; FAO and UNEP 2020). For instance, in this basin, the woody plants constitute the primary source of energy for domestic and artisanal uses (Mapenzi et al. 2023). The nexus of the high demand for wood energy, combined with slash‐and‐burn agriculture, is continuously threatening the forest resources, especially the woody plants (Makelele et al. 2022). This frequently results in the disappearance of useful woody species and particularly fragile species due to the excessive removal of their organs such as bark, roots, and stems (Badjaré et al. 2018). Therefore, to manage sustainably the natural resources, managers and decision‐makers need to have constant access to quality information on these resources (Rondeux 1994). Indeed, ethnobotanical studies play a crucial role in understanding the relationships between local communities and biodiversity by providing qualitative and quantitative data on plant species used for food, medicine, and cultural purposes. This traditional knowledge is essential for developing conservation strategies that integrate the needs of local populations with ecosystem protection while offering integrated management solutions for sustainable development (Monari et al. 2022; Tardío and Pardo‐De‐Santayana 2008; Phillips et al. 1994).

There is abundant literature on ethnobotanical studies that address aspects related to the assessment of the importance of vegetation in an ethnic group or in single and/or multiple forest habitats (Tardío and Pardo‐De‐Santayana 2008; Yarnvudhi et al. 2016; Prance et al. 1987; Phillips et al. 1994). In addition, some analyses have highlighted the medicinal and nutritional importance of woody species for local communities (Faruque et al. 2019; Chekole 2017; Islam et al. 2014; Sivasankari et al. 2014; Mirutse Giday et al. 2009). These findings have emphasized the necessity of ethnobotanical research to understand the shared relationships between humans and plants. However, as noted in the work of Prance et al. (1987), the utility of plant species is a function of ethnic group specificity and varies across communities. Thus, evaluating the use value of woody species at the community level is key for implementing appropriate conservation policies for these species at the landscape level.

Specifically, the Yangambi Biosphere Reserve (YBR) faces major challenges, such as deforestation, degradation of forest ecosystems, the depletion of animal species due to unsustainable hunting, and the intensification of unsustainable resource exploitation practices (van Vliet et al. 2018; van Vliet et al. 2019; Barbier 2007). These human activities have a direct impact on biodiversity, particularly threatening woody species. Moreover, the lack of scientific and socio‐economic information on the spatial distribution of useful woody species and their socio‐cultural value, combined with the absence of management policies specifically focused on multi‐use species, represent a major obstacle to the sustainable valorization and management of forest resources in the YBR.

This study attempts to address the gap in scientific knowledge about the diversity of the most useful species by analyzing their use value and assessing their abundance in the YBR landscape. The results will provide valuable insights into the socio‐cultural uses of these species in the Yangambi region to guide reforestation policies and forest management decisions. The findings will also contribute to broader conservation efforts by linking ethnobotanical knowledge with sustainable management strategies.

## 2 Materials & Methods

### 2.1 Location and Background

**FIGURE 1:**
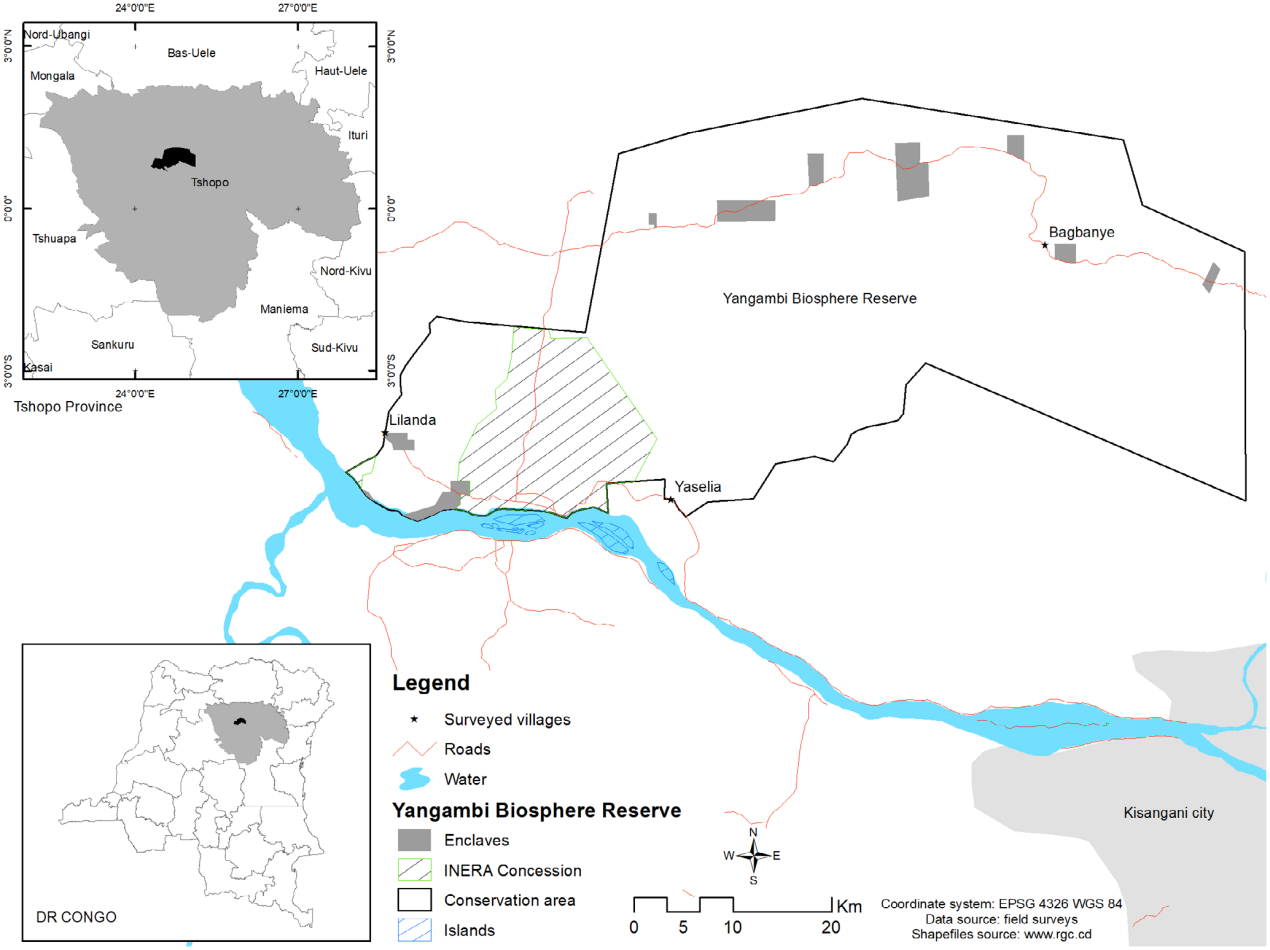
Location of the villages Yaselia, Lilanda, and Bagbanye in the YBR landscape within Kisangani town and INERA concession Situation of this landscape in Tshopo Province and in DRC.

This research is conducted in three villages located on the periphery of the Yangambi Biosphere Reserve (YBR): Yaselia, Lilanda, and Bagbanye (Figure 1). These villages were selected following the pre‐survey work, which classified them according to their demographics, cultural diversity, and age of settlement in the area. Administratively, Yaselia village is in the Yelongo grouping, and Lilanda village is in the Yambau grouping, all in the Turumbu sector, Isangi territory, while Bagbanye village is in the Bamanga grouping, Banalia territory. The Yangambi Biosphere Reserve (YBR) and all the villages are situated in the Tshopo province of the Democratic Republic of Congo (DRC), specifically between the geographical coordinates 24°18′ to 25°08′ East longitudes and 00°43′ to 01°08′ North latitudes, with altitudes varying between 400 and 500 m. The population in these villages is composed mainly of Turumbu and Bamanga. The Yangambi region, where the villages of Yaselia, Lilanda, and Bagbanye are located, is in the equatorial zone, dominated by dense evergreen rainforest, with an average annual rainfall of 1750 mm and an average temperature of 25°C. This landscape is characterized by its incredible biodiversity composed of evergreen monodominant forests dominated by *Gilbertiodendron dewevrei* (De Wild.) J. Léonard or *Brachystegia laurentii* (De Wild.) Louis ex Hoyle (Luambua et al. 2021). In addition, the YBR homes iconic species such as the *giant pangolin* ( *Manis gigantea* ), the *hippopotamus* ( *Hippopotamus amphibius* ), the *red river hog* ( *Potamochoerus porcus* ), and *cercopithecus monkeys* (*Cercopithecus spp*.) (Mpoyi et al. 2024; Kasongo et al. 2023).

Socio‐economically, the local community lives from agricultural activities, hunting, fishing, and collection of non‐timber forest products (Mangaza et al. 2021). Furthermore, activities such as transport, petty trade, and charcoal making constitute the economic tissue in these villages (Schure et al. 2019). However, many people are living under the poverty threshold, which explains their total dependence on natural resources, especially on woody plants for their foods, incomes, and medicines (Neema et al. 2022; Garrity et al. 2010). While the customary ruler on this topic seems not constrained due to the demographic explosion as well as the mix of people's cultures in this region, and consequently threatens the biodiversity conservation in this protected area landscape (Mpoyi et al. 2024; Kipute et al. 2023).

### 2.2 Ethnobotanical Survey Based on the Local Use of Woody Species

This process was started by a pre‐survey, which identified 29 woody species commonly used by the local population. Then, the ethnobotanical surveys were carried out in Bagbanye, Lilanda, and Yaselia to determine the use of these woody plants in this landscape. Toward this, the criteria of seniority in the village, age of respondents, social status, and representativity were applied to select the sample through these villages. Hence, the random sampling technique allowed for questioning respectively 29, 33, and 43 households in Bagbanye, Lilanda, and Yaselia, representing 10% of households per village. Indeed, this 10% was based on the statistics number of households obtained from local health services. This method ensures unbiased results and provides a representative sample, reflecting the diversity of ethnobotanical practices within the community. The survey focused on the respondents' knowledge of the species selected by village, their uses, the parts used, the areas of supply, and the management techniques applied to the various species. In this study, six uses had been identified regarding the local knowledge of woody plants, for example for construction, food, traditional medicine, trade, handicrafts, and energy wood. However, these six main categories of use also include minor uses such as for use 6 (wood energy), which includes planks used for cooking, as well as charcoal produced from these species.

In accordance with research ethics guidelines, including integrity and transparency, this study was conducted following scientific and ethical approval granted by the Pedagogical Council of the University of Kisangani, acting as the Scientific and Ethics Committee (Ref. No. 023/UNIKIS/FS/KGP/DFS/2018), as well as authorization from the Director of the National Institute for Agronomic Studies and Research (INERA). Regarding the individuals interviewed, we ensured that their free, prior, and informed consent was obtained while guaranteeing the anonymity and confidentiality of the information collected.

### 2.3 Ethnobotanical Index of Multiple‐Use Species

To assess the value of the species commonly used in Bagbanye, Yaselia, and Lilanda villages, five ethnobotanical indexes and a similarity index are calculated based on the information of the respondents (Ogwu et al. 2024; Tardío and Pardo‐De‐Santayana 2008; Reyes‐garcía et al. 2006; Prance et al. 1987). It is about the following:

#### 2.3.1 Number of uses (NU) per species

This index is calculated using the formula below (1), where NC is the number of use categories and NU is the sum of all categories for which the species is considered useful (Ogwu et al. 2024; Prance et al. 1987),

(1) $$\mathrm{NU}=\sum_{u=1}^{n}\mathrm{NC}$$

#### 2.3.2 Relative importance (RI) index

The relative importance index analysis was chosen in this study to rank species according to their relative importance. The RI Formula (2) below is used to determine this index.

(2) $$\mathrm{RI}=\frac{\text{RFCs}\;\left(\max\right)+\text{RNUs}\;\left(\max\right)}{2}$$

with RFCs being the relative frequency of citation over the maximum value of all species and RNUs being the relative number of use categories per species (Whitney 2021).

#### 2.3.3 Use value (UV) index

Use value is calculated for individual species to provide a quantitative measure of their relative importance to informants in an objective manner (Katayi et al. 2023). It is obtained through the following formula:

(3) $$\mathrm{UV}=\frac{\sum_{i}{\mathrm{UV}}_{\text{is}}}{\mathrm{ns}}$$

In this formula, UV refers to species' use value, UV_is_ refers to the number of use reports cited by informants for that plant species, and ‘ns’ refers to the total number of informants. The species with a high UV_is_ is the most used by local communities for different utilities (Ogwu et al. 2024; Islam et al. 2014).

#### 2.3.4 Cultural value for ethno‐species (CVe)

This index is one of three proposed to evaluate the species' importance in cultural, practical, and economic dimensions. The Formula (4) below allows the calculation of this cultural value index.

(4) $${\mathrm{CV}}_{e}={\mathrm{Uc}}_{e}\times {\mathrm{Ic}}_{e}\times {\text{ΣIUc}}_{e},$$

where Uc_e_ is the number of reported uses for the species divided by all potential uses of the species considered in the study. Ic_e_ is the number of informants who listed the species as useful divided by the total number of informants. IUc_e_ refers to the number of informants who mentioned each use of the species divided by the total number of informants (Reyes‐garcía et al. 2006).

#### 2.3.5 Fidelity level (FL) per species

It expresses the preference for one species over others. It is a way of calculating the percentage of informants who use a plant for the same purpose relative to all uses of all plants (Ogwu et al. 2024; Prance et al. 1987).

(5) $$\mathrm{FL}=I_{p}/I_{u}\times 100,$$

where *I*_*p*_ is the number of informants who use a species for a particular category of utility, while *I*_*u*_ is the number of informants who use the same t species for another category of utility.

#### 2.3.6 Jaccard similarity index

It allows a comparison between two sites because it evaluates the similarity between two surveys by taking the ratio between the species common to both surveys and those specific to each survey. It is calculated through the following Formula (6):

(6) $$I=\mathrm{Nc}/\left(\text{N1}+\text{N2}-\mathrm{Nc}\right).$$

In this formula, Nc is the number of species found in the two environments. While N1and N2 are respectively the number of species present in village 1 and 2. The index varies from 0 to 1. When this index is high, a significant number of species are found in both habitats, suggesting that inter‐habitat biodiversity is low (similar environmental conditions between habitats). In the opposite case, if the index decreases, only a few species are present in both habitats. Thus, when the species for the two habitats compared are completely different, this indicates that the different habitat conditions determine a high species turnover (Faruque et al. 2019).

However, respondents' responses on the appreciation of a use category are coded from 0 to 1. The score is 0 when the person does not use the species for any of the uses, and the score is 1 when the species is used for a specific use (Faruque et al. 2019).

Statistical analysis of the indices is performed through the “ethnobotanyR” package previously installed in the R software (R Core Team 2021; Whitney 2021). The latter allows the cultural importance of plant species to be assessed based on informant consensus.

### 2.4 Quantitative Inventory of Multiple‐Use Species

A preliminary survey was conducted among village elders to establish a comprehensive list of species deemed valid within the landscape. Based on this community consultation, 29 species commonly used by local communities were identified and validated. These species were subsequently included in the quantitative inventory to assess their distribution and abundance across the landscape.

To capture the ecological diversity of the region and ensure representative data, three key ecosystems were selected for the inventory: mature forest, secondary forest, and fallow land (Luambua et al. 2024, 2021). These ecosystems were chosen based on their prevalence in the landscape and their varying levels of human disturbance, which influence the availability and abundance of useful species. Mature forests represent relatively undisturbed habitats with long‐established tree populations, secondary forests reflect areas undergoing natural regeneration after disturbance, and fallow lands illustrate transitional zones shaped by shifting agriculture, where some useful species thrive due to their adaptability to disturbed environments.

**FIGURE 2:**
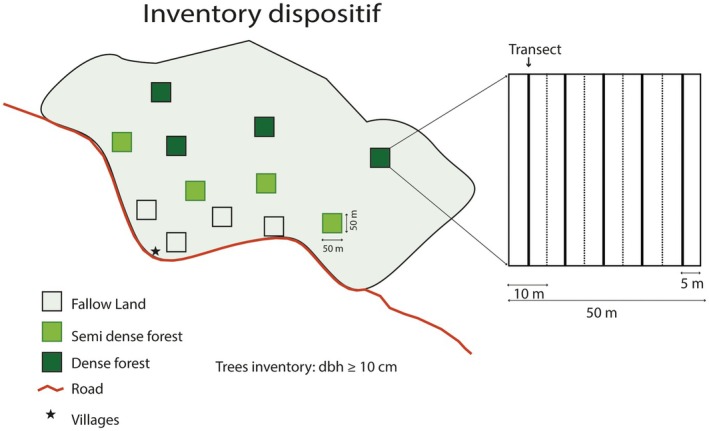
Forest inventory system applied in villages in the Yangambi landscape.

The inventories were conducted across these ecosystems in each village surveyed, using 4 plots of 50 × 50 m per ecosystem (1 ha per ecosystem per village, Figure 2). Plots were spaced 500 m apart to minimize spatial autocorrelation and capture variation within the landscape. This design ensured robust estimates of species abundance and allowed meaningful comparisons between ecosystems.

The boundaries of the plots were marked by opening the transects with a compass. These boundaries were also georeferenced using a Garmin 64× GPS. Then, all trees of the target species with a diameter at breast height (dbh), measured at 1.30 m above the ground, equal to or exceeding 10 cm, were identified and measured. This threshold (dbh ≥ 10 cm) is a standard convention in tropical forest inventories (Phillips et al. 2009) and is used to focus on mature trees that significantly contribute to the forest structure.

This inventory aims to provide information on the availability of useful species in this landscape due to the pressure exerted on these resources through the habitats surrounding the villages and the threat that this exploitation represents for the YBR if these useful species are absent within the village land. The availability of each species was expressed in terms of the number of stems per hectare for food and medicinal uses. For uses relating to woody biomass, the availability of the species was expressed in terms of biomass. The measured dbh data were essential for estimating tree height (*H*) (Hubau et al. 2020) and then the biomass stock of a species in the forest using well‐established allometric equations (Chave et al. 2014; Hubau et al. 2020).

$$H=a\times {\left(1-e^{-b\times D}\right)}^{c}$$

where *H* is the total tree height (in meters); *D* is the diameter at breast height (DBH) or diameter at 1.3 m (in cm); the three coefficients *a* = 50.8, *b* =0.0499, and *c* = 0.706 are specific to the terra firme forests in the Eastern Congo Basin and East Africa, as defined by Hubau et al. (2020); and *e*is the base of the natural logarithm.

$$\mathrm{AGB}=0.0673\times {\left({\mathrm{\rho D}}^{2}H\right)}^{0.976}$$

with AGB being aboveground biomass (in kg), *ρ* being the wood density (in g/cm^3^), *D* being the diameter at breast height (DBH) or diameter at 1.3 m (in cm), and *H* being the total tree height (in m).

The inventories covered a surface area of 9 ha, corresponding to 3 ha per village (1 ha per ecosystem). The locations of these plots were strategically chosen using forest cover data extracted from 2020 Landsat 7 satellite images (Kearsley et al. 2013). All survey and inventory data were encoded in an Excel sheet. Then, the R software (R Core Team 2021) were used to calculate the indices and produce the graphs, as well as to analyze the variance of species abundance in different habitats.

This approach accounted for ecological gradients and aligned with the broader goal of understanding how different land uses influence the distribution of species critical to local livelihoods. By bridging ecological and ethnobotanical perspectives, the study provides valuable insights into the relationship between biodiversity and community resource use.

## 3 Results

### 3.1 Useful Woody Species in Yangambi Landscape

Forest resources (timber and NTFP) are among the main pillars of livelihoods for forest communities. This study emphasizes the useful common species and assesses their use and cultural value in Yaselia, Lilanda, and Bagbanye villages, within the YBR.

In Lilanda and Yaselia village, the 29 multipurpose species identified during the ethnobotanical survey belong to 19 families. The Fabaceae family is the most represented, with 7 species in each village. In Lilanda, it is followed by Meliaceae, Euphorbiaceae, Apocynaceae, and Cecropiaceae, with two species each. While in Yaselia, the Euphorbiaceae family follows with three species; then there are the Burseraceae, Moraceae, and Annonaceae, with two species each. As for Bagbanye village, the 29 species belong to 15 families, with Fabaceae represented by six species, Meliaceae with four species, Moraceae with three species, and Burseraceae, Euphorbiaceae, and Myristicaceae with two species each.

This ethnobotanical survey revealed that the Fabaceae family abounds in a significant number of multipurpose species in the villages investigated in the Yangambi region. A comparative analysis of Jaccard's index between Yaselia and Lilanda villages gave a value of 0.43 (i.e., 43% similarity of species between the two villages), and a value of 0.40 between Lilanda village and Bagbanye and finally 0.5 between Yaselia and Bagbanye. Jaccard's similarity coefficient shows that floristic similarity between the three villages is low. This means that each of these villages is characterized by a specific flora assemblage that it considers to be of great use and cultural value.

Furthermore, the study of the use value of woody species is crucial to determine their socio‐cultural importance in a community. Thus, the analysis of the use value of the species identified by the community revealed that a certain category of species stands out from the others in terms of the number of uses, cultural value, and use value.

In Lilanda village, for example, species such as *Entandrophragma cylindricum* (Sprague) Sprague & Hoyle (UV 2.39), *Petersianthus macrocarpus* (P.Beauv.) Liben (UV 2.15), *Ricinodendron heudelotii* (Baill.) Pierre ex Heckel (UV 2.15), *Scorodophloeus zenkeri* Harms (UV 2.09), and *Pentaclethra macrophylla* Benth. (UV 1.88) obtained a high use value compared to other species in the same village. While in Yaselia village, species such as *Petersianthus macrocarpus* (UV 2.21) and *Entandrophragma cylindricum* (UV 2.19) are the only ones to obtain a use value higher than 2. Also, in the top 5, there are *Uapaca guineensis* Müll.Arg. (UV 1.77), *Blighia welwitschii* (Hiern) Radlk. (UV 1.72), and *Chrysophyllum lacourtianum* De Wild. (UV 1.67). Finally, in Bagbanye, the species with high use value are *Dacryodes edulis* (G.Don) H.J. Lam (1.885), *Petersianthus macrocarpus* (UV 1.81), *Gilbertiodendron dewevrei* (De Wild.) J. Léonard (UV 1.65), *Entandrophragma angolense* C.DC. (UV 1.58), and *Uapaca guineensis* (UV 1.5).

**FIGURE 3:**
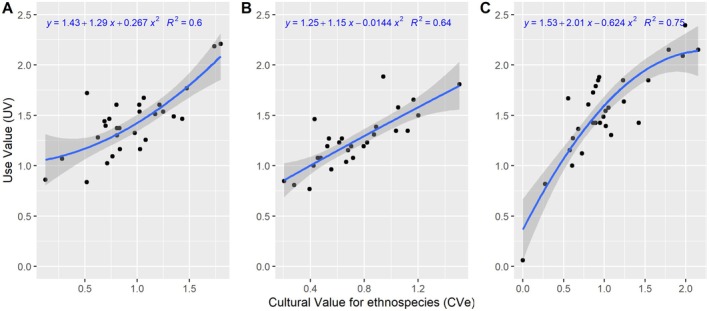
The relationship between the cultural value and use value of plant species in three villages within the Yangambi region: Yaselia (A), Bagbanye (B), and Lilanda (C). The cultural value of each species reflects its significance to local communities in terms of traditional practices, rituals, and social customs, while the use value indicates the frequency and range of practical uses the species has, such as food, medicine, construction, and other daily needs.

However, analysis of the correlation between these species' cultural and use values gives a coefficient of determination of 0.60 for Yaselia, 0.75 for Lilanda, and 0.64 for Bagbanye. This means that the use value of these species is closely linked to their cultural value. Species with high use value have cultural, practical, and economic importance for local communities (Figure 3).

### 3.2 Abundance of Multipurpose Species in the Village Harvest Area

**FIGURE 4:**
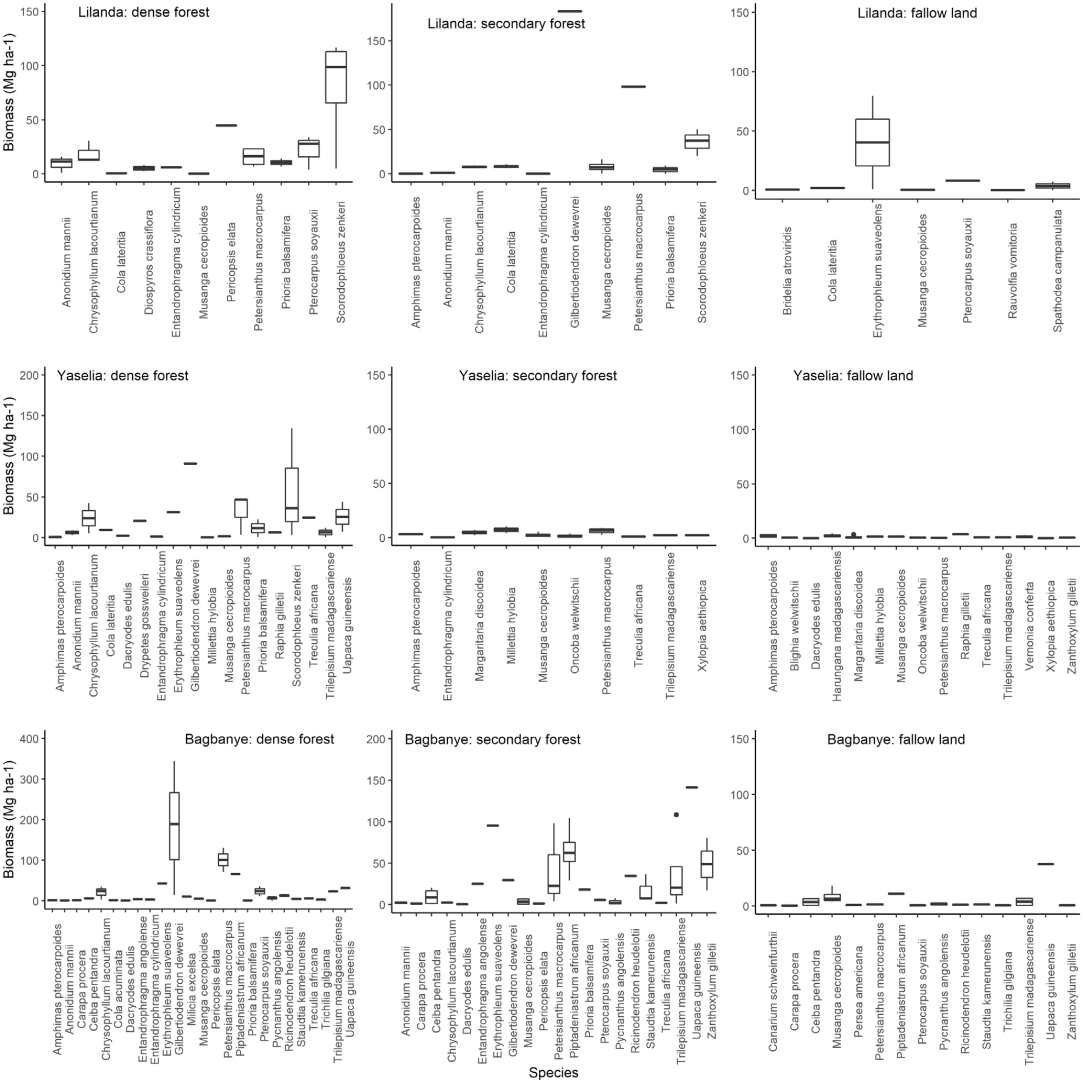
Abundance of plant species inventoried in the village farming areas of three villages: Yaselia, Lilanda, and Bagbanye, within the Yangambi region. The abundance of species is measured by the frequency and biomass of species found in each village's farming landscape, including habitats such as dense forest, secondary forest, and fallow lands.

The quantitative inventory of species recognized for multiple uses by communities revealed that some species, although important for community needs, are no longer present in the village harvesting area. Figure 4 presents the biomass of species in the inventory plots installed in mature and secondary forests and fallow land in each village.

Through this selective forest inventory of multipurpose species, we notice that in Lilanda village, not all 29 species are present in the sample area and that species such as *Scorodophloeus zenkeri*, *Pericopsis elata* (Harms) Meeuwen, and *Pterocarpus soyauxii* Taub. are those with an abundant biomass in the dense forest. In the secondary forest, we find *Gilbertiodendron dewevrei*, and *Petersianthus macrocarpus*. On the other hand, in the fallow, there is *Erythrophleum suaveolens* (Guill. & Perr.) Brenan, a species which is the most represented. However, among the species that are not present in the inventory plots but that had a high use value for the Lilanda people, we have *Ricinodendron heudelotii*, *Pentaclethra macrophylla* , *Guarea cedrata* Pellegr. ex A.Chev., and *Dacryodes edulis* .

By performing an analysis of variance of the abundance/biomass of species in the three habitats, we find a non‐significant difference between the biomass of species in primary and secondary forest and between secondary forest and fallow. In contrast, the biomass of species in the fallow is significantly lower than that found in the dense forest (Kruskal–Wallis, *p* = 0.019).

**FIGURE 5:**
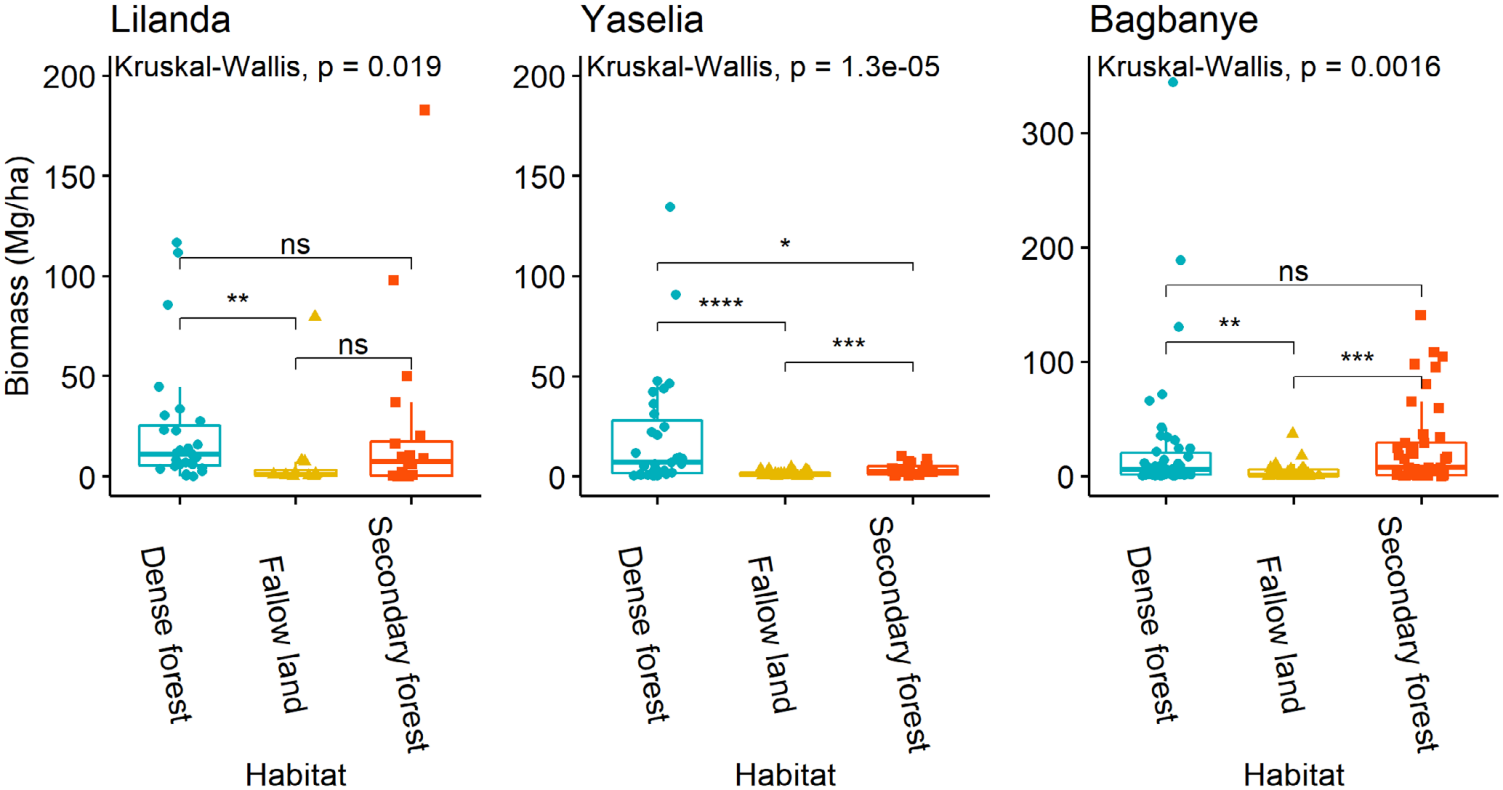
Analysis of variance (ANOVA) comparing the biomass of plant species in three different habitat types: Dense forest, secondary forest, and fallow land in the villages of Lilanda, Yaselia, and Bagbanye, within the Yangambi Biosphere Reserve. The biomass data represent the total plant material (aboveground) per unit area in each habitat type. It highlights the significance of dense forests, the intermediate state of secondary forests, and the low biomass of fallow lands, reflecting the ecological status and anthropogenic pressures in the Yangambi region. * (*p* < 0.05): indicates statistical significance at the 5% level; ** (*p* < 0.01): indicates statistical significance at the 1% level; *** (*p* < 0.001): indicates very strong statistical significance at the 0.1% level; ns: indicates no significant differences.

In Yaselia village, the primary forest has significantly different biomass than two other habitats, including secondary forest and fallow. In addition, only 11 and 16 species out of 29 targeted were found in secondary forest and fallow, respectively. As for Bagbanye village, the trend is different from the other villages, between the dense and secondary forests where the biomass is not different. In addition, these two areas show a significant difference in biomass with the data from the fallow (Figure 5).

**FIGURE 6:**
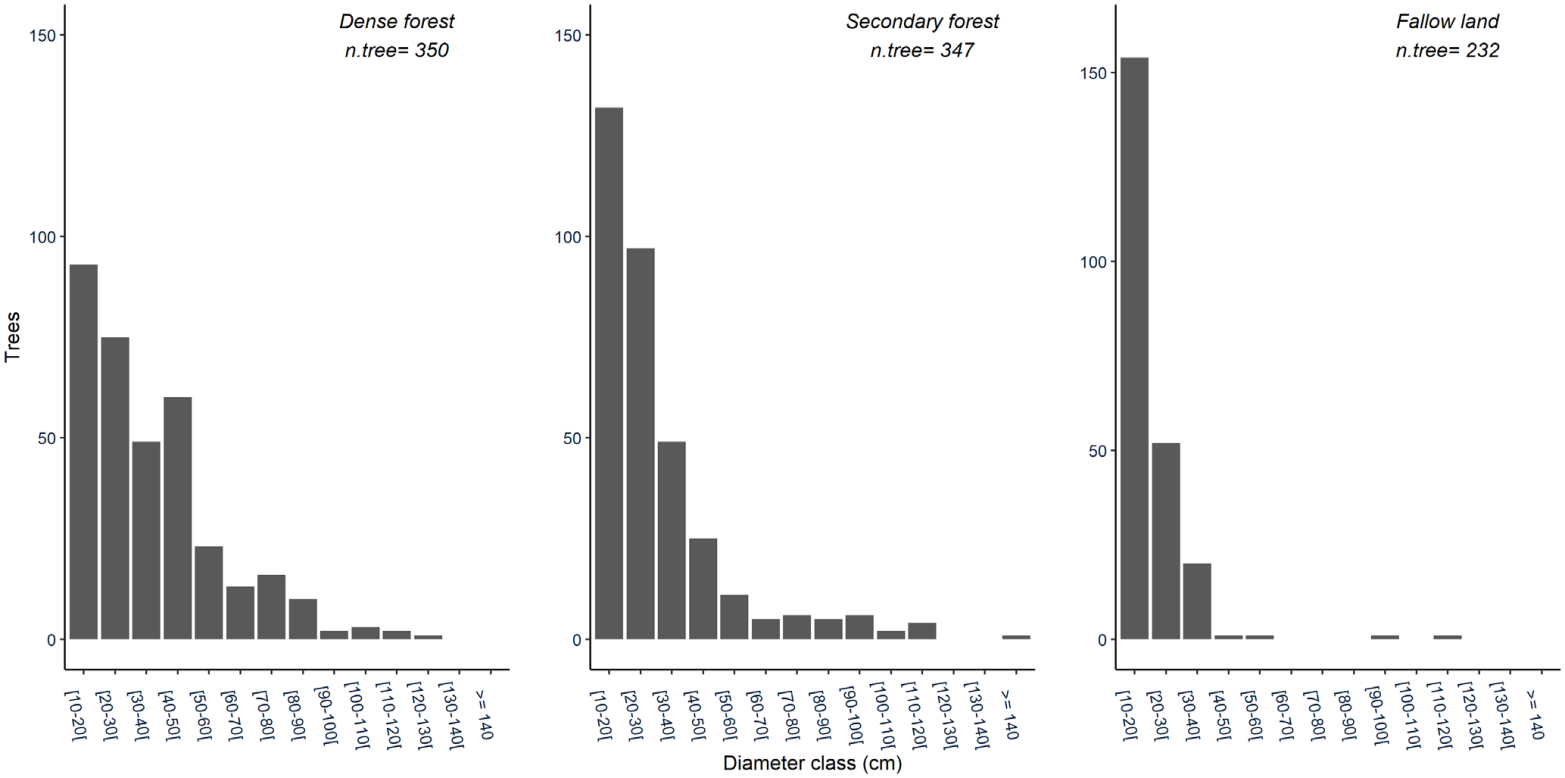
The diameter structure (DBH—diameter at breast height) distribution of multipurpose species in dense forest, secondary forest, and fallow land. The diameter structure indicates the range of tree sizes present in each habitat, highlighting the distribution of different age classes and the presence of both small‐ and large‐diameter trees.

The analysis of the diameter structure in a stand allows one to know the regeneration of a given species. It remains a major prerequisite for the implementation of new management policies to ensure their sustainability, not only for future generations, but also to satisfy current needs. The Figure 6 presents the diameter classes to highlight the abundance of these useful species inventoried through three ecosystems, namely, primary forest, secondary forest, and fallow land.

The distribution of species in function of diameter classes reveals that the three habitats have decreasing or inverted J‐shaped diametric structures, where the number of stems decreases as the diameter increases. However, the village forests of Yaselia, Lilanda, and Bagbanye are still dominated by small‐diameter trees. The 10–20 cm, 20–30 cm, and 30–40 cm classes are highest in secondary forest and fallow land. As for the primary forest, the 40–50 cm diameter class also recorded a high number of species.

This study found that there was a significant decrease in the multipurpose species in fallow land and secondary forest. Compared with dense forest, analysis of the variance between these three habitats reveals a highly significant difference (*p*‐value < 2e‐16). This means that the village farming areas closest to dwellings (fallow land and secondary forest) are subject to strong pressure from communities, and the impact of these human activities is reflected in a reduction in species diversity, but also in the abundance and/or biomass of the species present in the environment.

### 3.3 Use Value of Species in Yangambi Landscape

Calculating the fidelity level (FL) index in an ethnobotanical study gives an idea of the main uses of a species by the local community. The FL is designed to quantify the importance of a species for a given purpose.

**FIGURE 7:**
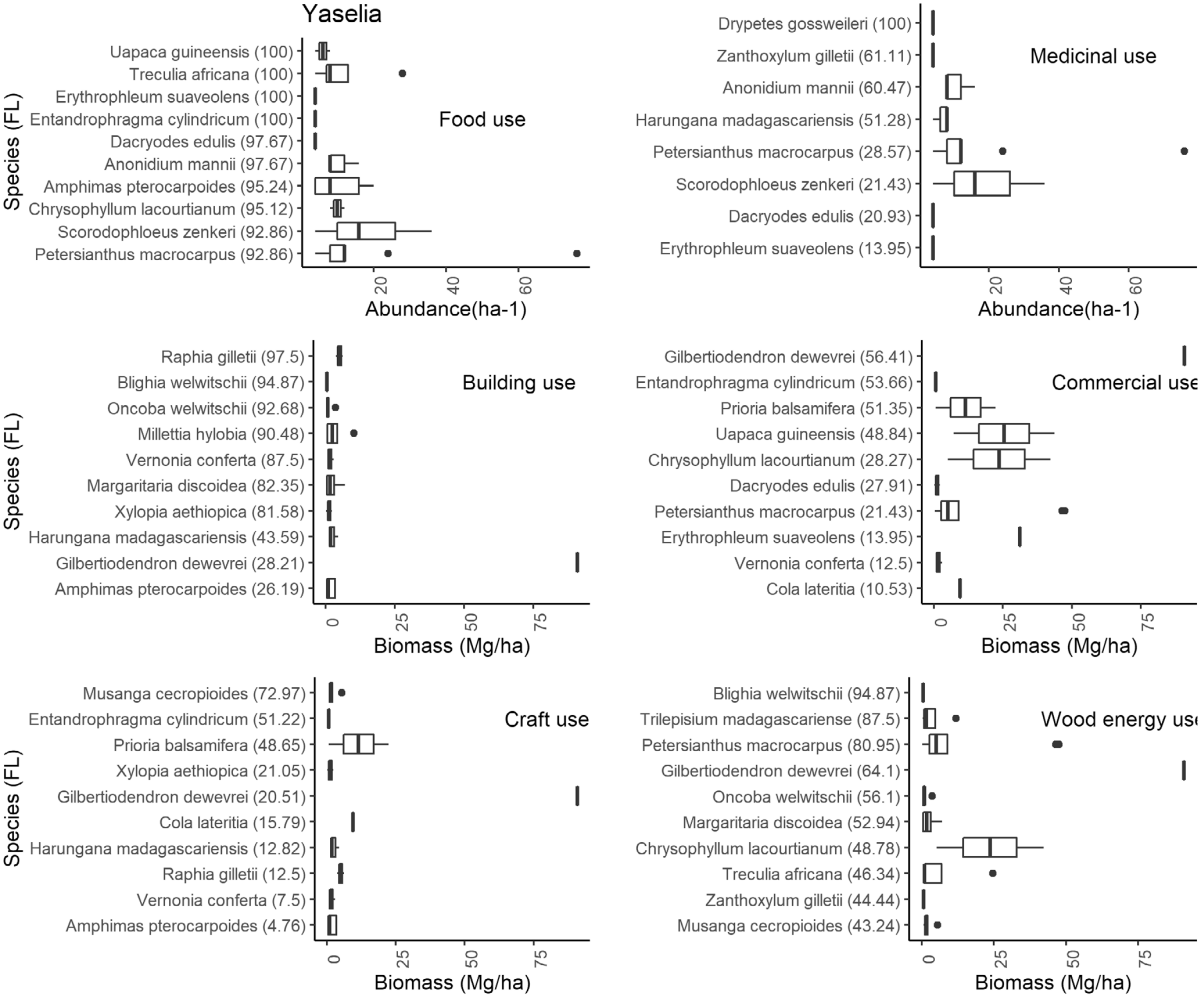
The fidelity level (FL) and abundance/biomass of the top 10 most used species in Yaselia village, categorized by their primary use type (food, medicine, construction, wood energy, craft, and commercial). Fidelity level (FL) is a measure of the species' degree of cultural significance, indicating how frequently a species is associated with a particular use category. This figure provides an overview of the relationship between the ecological availability of species and their cultural importance in the context of the village of Yaselia.

A maximum FL indicates the frequency and high use of the species for a particular purpose by informants in the study area. In our case, this index gives the names of the most frequently cited species by category of use. The Figure 7 illustrates the species ranked in order of importance, considering the FL (see data in brackets) of species in function of a specific use.

One category of species stands out for the way they are used by Yaselia communities for different purposes. For food, for example, several species are used 100%: *Uapaca guineensis*, *Treculia africana* Decne. ex Trécul, *Erythrophleum suaveolens* , and *Entandrophragma cylindricum*. *Drypetes gossweileri* S.Moore is renowned for its medicinal properties, followed by *Zanthoxylum gilletii* (De Wild.) P.G. Waterman. As far as construction is concerned, species such as *Raphia gilletii* Becc. (whose branches are used to cover houses), *Blighia welwitschii*, *Oncoba welwitschii* Oliv., and *Millettia hylobia* Louis ex Hauman are the most widely used by the Yaselia population.

**FIGURE 8:**
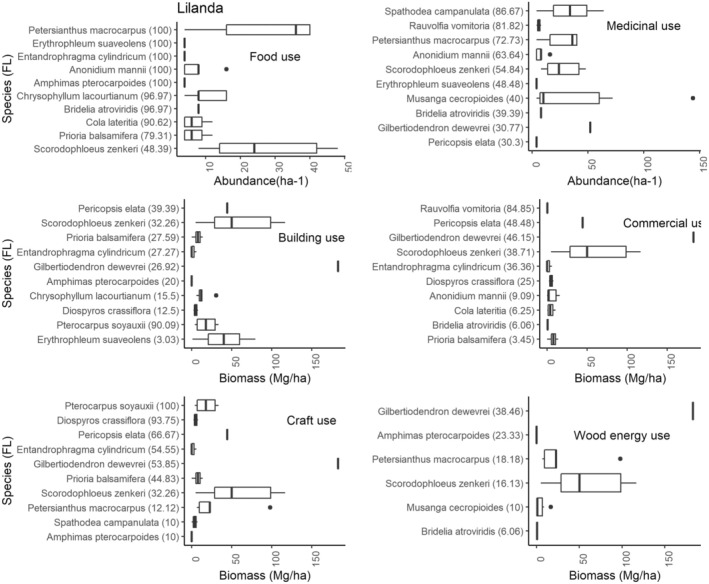
The fidelity level (FL) and abundance/biomass of the top 10 most used species by use category in the village of Lilanda. This figure clearly describes how the fidelity level (FL) and abundance/biomass interact to show both the cultural importance and ecological availability of plant species in Lilanda village, facilitating the understanding of the community's reliance on these species and their potential for sustainable management.

However, species such as *Erythrophleum suaveolens* , *Petersianthus macrocarpus*, *Entandrophragma cylindricum*, *Harungana madagascariensis* Poir., *Gilbertiodendron dewevrei*, and *Chrysophyllum lacourtianum* are cited in more than three use categories at Yaselia. This testifies to their usefulness for the survival of the local population. However, some species cited by the communities for a specific use are not present in the inventory plots, and those cited for construction and handicrafts are not abundant in the village exploitation zone. The Figure 8 shows the FL and abundance of species in the village of Lilanda.

In Lilanda, species such as *Petersianthus macrocarpus*, *Entandrophragma cylindricum*, *Erythrophleum suaveolens* , *Scorodophloeus zenkeri*, and *Gilbertiodendron dewevrei* have a great cultural and economic value. They are used in more than four use categories. However, the biomass of species used for charcoal production has declined. The *Pterocarpus soyauxii* species is the most widely used for handicrafts, its main use being the crafting of pirogues.

**FIGURE 9:**
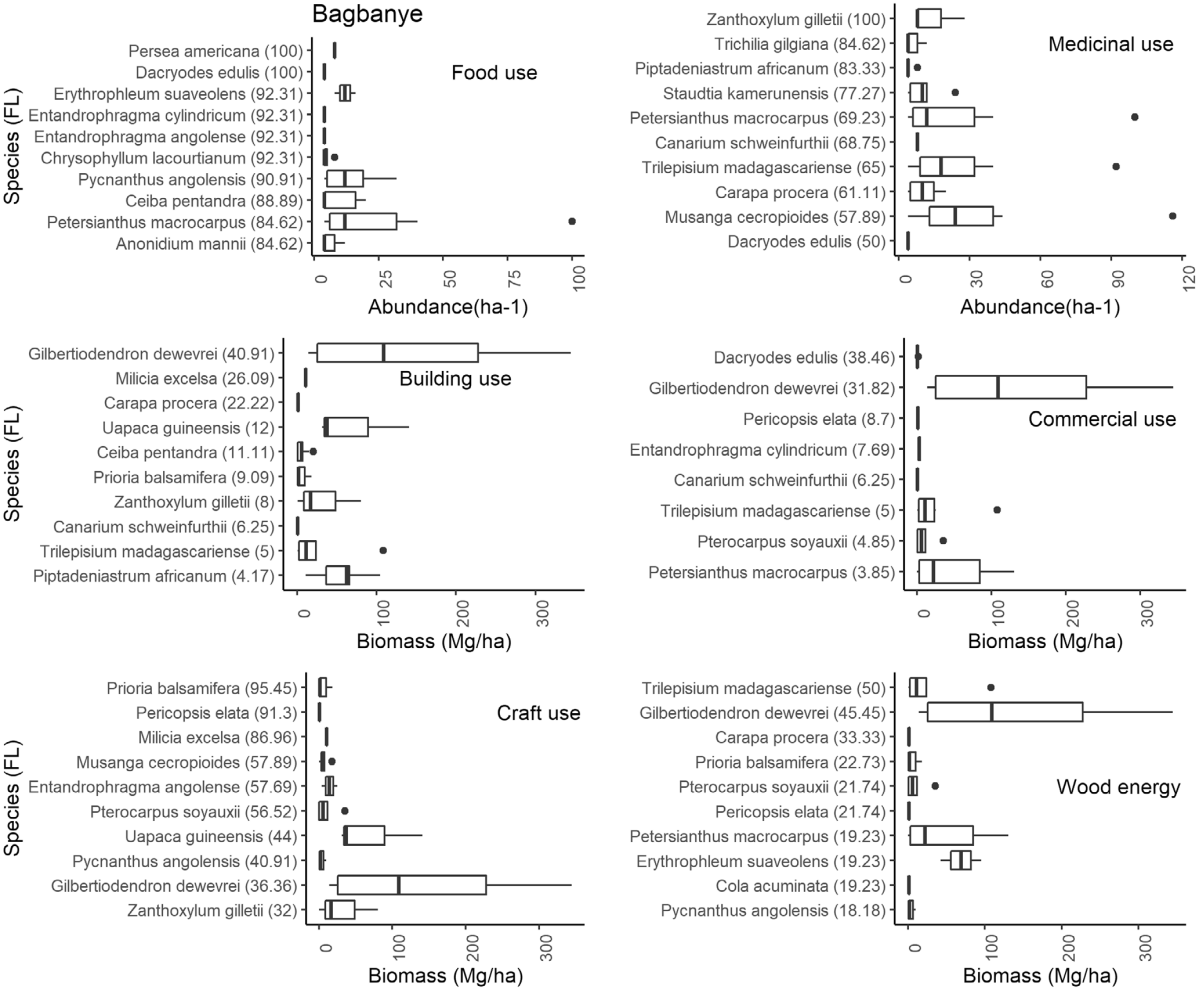
The relationship between fidelity level (FL) and abundance/biomass of the top ten most used species by use category in the village of Bagbanye. This combination of FL and abundance/biomass highlights species that are both essential to local needs and available in sufficient quantity, providing valuable insights for resource management, conservation, and sustainable use of these multi‐purpose species in the Yangambi region.

Species such as *Petersianthus macrocarpus*, *Erythrophleum suaveolens* , *Entandrophragma cylindricum*, and *Gilbertiodendron dewevrei* have a vital importance in the Bagbanye community, as they are used for construction, food, medical treatment, and, in some cases, charcoal production. Unfortunately, their abundance is low in the areas where the villages are exploited (Figure 9).

In many cases, the food species cited by the communities are angiosperms, that is trees whose fruit is edible. For medicinal use, the fruit, bark, leaves, and roots are the parts used for this purpose. In summary, the species most cited by the communities for multiple uses in all the villages have a low density, and others have a low biomass. As a result, the interviews with the communities revealed that no provision has been made in all the villages for the effective and sustainable management of these species. Their regeneration is left to a natural effect, despite their cultural and socio‐economic importance.

## 4 Discussion

### 4.1 Woody Species and Their Uses in the Yangambi Landscape

The ethnobotanical survey conducted in the villages of Yaselia, Lilanda, and Bagbanye revealed that the 29 species selected by the village to identify their usefulness belong to 19 families in the villages of Yaselia and Lilanda and 15 families in the village of Bagbanye, with a predominance of Fabaceae which, according to Toirambe et al. (2009), ranks first among the families with high relative importance in the Yangambi landscape. This predominance of Fabaceae is also observed in the flora of Ethiopia (Eshete and Molla 2021) and in the dense forests of Africa (Ogwu et al. 2024; Miabangana and Malaisse 2020; Faruque et al. 2019). Furthermore, the forest inventory revealed that species from the Fabaceae family, as well as Euphorbiaceae, Meliaceae, Moraceae, and Burseraceae are among the most abundant families in the sampled village forests. These results are similar to those obtained in the sacred forests of the village of Batoufam, in eastern Cameroon, where more than 50% of the total trees with high use value were represented by these four families (Noumi 2011). Additionally, these five most abundant families in the inventory plots are among the 10 families that contribute to half of the species richness of tropical forests (Dewalt et al. 1999; Gentry 1988), as well as the families of species with high use value in the forests of the Bolivian Amazon (Reyes‐garcía et al. 2006).

However, we noted that 21% of the species cited in Yaselia are used for charcoal production, compared with 8% in Lilanda and 4% in Bagbanye. This can be explained by the fact that the inhabitants of Lilanda do not produce charcoal because of the distance between the village and the town of Kisangani, which would require more labor. This influence of the distance between the production area and the place of sale has been demonstrated in the work of Schure and Lwanga (2018); Schure et al. (2013) on the level of charcoal production, which decreases as one moves away from the major towns. Nevertheless, charcoal production is not common in Bagbanye and Lilanda. The population more often practices agriculture, hunting, and gathering to meet their subsistence needs, a characteristic of the forest peoples of Central Africa (Joiris 2000).

In addition, the calculation of the use value revealed that species such as *Entandrophragma cylindricum*, *Petersianthus macrocarpus*, *Ricinodendron heudelotii*, *Scorodophloeus zenkeri*, *Uapaca guineensis, Dacryodes edulis, Gilbertiodendron dewevrei*, and *Raphia gilletii* obtained a use value ≥ 2. These species also have a high cultural value (CVe) and contribute to the well‐being of local populations. As far as food is concerned, *Dacryodes edulis, Erythrophleum suaveolens, Entandrophragma cylindricum, Petersianthus macrocarpus*, and *Treculia africana* are the most used species by the communities. These plants, primarily valued for their edible fruits, play a crucial role in the nutrition of local populations. Their significance extends beyond just dietary benefits, as they also hold deep social and cultural value. These species share many similarities with those identified in other ethnobotanical studies, both in terms of diversity and cultural importance (Mboujda et al. 2024; Bieski et al. 2015; Omonhinmin 2014).

For medicinal use, the species commonly used by the communities are *Zanthoxylum gilletii, Drypetes gossweileri, Spathodea campanulata* P.Beauv., *Trichilia gilgiana* Harms, and *Piptadeniastrum africanum* (Hook.f.) Brenan, while the stems, leaves, and roots are the parts used for medicinal purposes. In most cases, the same species are used for construction, trade, firewood, and handicrafts, and according to interviews with communities, the most used species are *Pericopsis elata, Gilbertiodendron dewevrei, Carapa procera* DC., *Milicia excelsa* (Welw.) C.C.Berg, and *Entandrophragma angolense*. Indeed, studies by Dean (2024), Katayi et al. (2023), Willy et al. (2023), Eshete and Molla (2021), Singh et al. (2020), and on tropical African plants have documented the diverse applications of these species, underscoring their vital role in agricultural practices and sustainable resource management. These studies highlight the multifaceted importance of these plants to local communities, contributing not only to health care but also to economic stability and environmental sustainability. For example, in Central Africa, species like *Ricinodendron heudelotti* (Euphorbiaceae), *Entandrophragma cylindricum* (Meliaceae), and *Dacryodes edulis* (Burseraceae) are highly valued for their medicinal and nutritional uses. In the Amazon, *Hevea brasiliensis* (Euphorbiaceae) is a major source of rubber, while *Mauritia flexuosa* (Moraceae) is used for its fruit and medicinal properties (Reyes‐garcía et al. 2006).

It was found that the main parts of these species, which are the most widely exploited, are the stems for construction, trade, crafts, and energy wood, followed by the fruits (seeds), mainly for food. These two main uses demonstrate the importance of these species for food and trade. Indeed, the high demand for these organs for domestic consumption and trade is leading people to overexploit them (Abdourhamane et al. 2017). Furthermore, the most exploited plant organs are from natural regeneration in the forest. Unfortunately, observation in the field and interviews with communities have revealed that these species, which have great use and cultural value, are not specifically managed. Uncontrolled use of woody species would lead to a reduction in timber resources and the species most used for vital needs such as food and medicine (Dean 2024). Consequently, communities must travel long distances in the forest to harvest non‐timber forest products (Kipute et al. 2021). Faced with this situation, it is necessary to manage these resources sustainably to avoid the total disappearance of some sensitive species. For example, in some regions of South America, the harvesting of fruits and wood for construction is regulated to ensure the natural renewal of resources (Kunwar et al. 2013). In the case of RBY, it would be more effective to train local communities in sustainable harvesting methods, assisted regeneration of the most exploited species, and raise awareness about best practices for forest resource management. This would not only ensure the availability of forest products in the short and long term but also secure the livelihoods of both current and future communities.

### 4.2 Abundance of Multi‐Use Species in Villages

For a community, a tree's uses motivate its exploitation and/or preservation (Gautier 1994). However, the lack of information on the dynamics of woody species is a constraint that limits the development of participatory and sustainable forest management strategies. The inventory of multi‐use species carried out in the village forests of the Yangambi region identified a total of 948 stems over 9 ha, giving an average of 105.22 stems/ha. Primary and secondary forest each accounted for 37.59% of all the stems inventoried, compared with 24.82% in fallow land.

Based on diameter (DBH), *Ricinodendron heudelotii*, found in the secondary forest of Bagbanye village, had the largest diameter, at 140.7 cm. *Gilbertiodendron dewevrei* followed this at 124.6 cm and *Piptadeniastrum africanum* at 118.7 cm, both found in the primary forest of Bagbanye village. In addition, the most predominant species in the study site according to their frequency of appearance in the plots was *Musanga cecropioides* R.Br. ex Tedlie (206 records, or 21.73% of the total record). It is very present in fallow land and secondary forests, followed by *Petersianthus macrocarpus* (123 records or 12.97%), *Trilepisium madagascariense* DC. (72 records or 7.59%), *Scorodophloeus zenkeri* (62 records or 6.54%) and in fifth place by *Gilbertiodendron dewevrei* with 56 records (5.91%). However, in terms of biomass, *Gilbertiodendron dewevrei* has the highest biomass in the study area, followed by *Petersianthus macrocarpus, Scorodophloeus zenkeri, Piptadeniastrum africanum*, and *Uapaca guineensis*. Undoubtedly, these results align with the trend observed in African tropical forests, where large, long‐lived species significantly dominate the biomass of forest ecosystems and play a crucial role in ecological stability and carbon sequestration (Mboujda et al. 2024; Faruque et al. 2019; Eshete and Molla 2021).

The analysis of the variance in the three habitats (primary forest, secondary forest, and fallow land) revealed that the primary forest is the best represented in terms of abundance and biomass of multi‐use species. The fallow land is the least diversified and is dominated by small‐diameter trees. We therefore believe that the proximity of fallow land to residential areas, combined with the socio‐economic importance of multi‐use species, would have led to their over‐exploitation and resulted in their reduction in areas on the outskirts of villages. Therefore, the most used species by communities for their livelihoods are located through the forest edges or forests adjacent to villages (Yarnvudhi et al. 2016). These findings reflect the communities' interest in these species and should be supported by raising awareness of best management practices for multiple‐use species.

According to Abdourhamane et al. (2017), the impact of pressures on plant species is generally assessed through the demographic structure of stands. In the present study, the tree structure shows that small‐diameter trees dominate the forests in the villages investigated and that there is a virtual absence of stems in certain diameter classes, which is evidence of these pressures. Qualified as a decreasing exponential distribution by Pascal (2003), this structure of stands with a high density of small‐diameter classes guarantees the future of tree populations, while the large trees, although at a low density of classes, ensure the continuity of the standby acting as seed trees. Such a distribution is a sign of stability and good natural regeneration.

However, it should not be forgotten that the strong interest in the species as a source of food, medicine, firewood, and income is likely to increase the demand for products and, therefore, the vulnerability of the multipurpose species. Moreover, the exploitation of various parts of the trees, including bark stripping and root harvesting, reduces the vitality of the trees, compromising their ability to regenerate naturally and thus accelerating their decline. The studies by Cruz et al. (2021), Bodeker et al. (2014), Muler et al. (2014), and Wadt et al. (2008), conducted in the Brazilian Amazon and Central Africa, highlight that the excessive exploitation of roots, bark, and fruits has direct consequences on the survival of certain species. Thus, an overall analysis of the vegetation structure could conceal a degradation process affecting certain species with high use and socio‐economic value. This is what we found in the forest of the villages studied, where taken individually, some species of high use value have either disappeared or are poorly represented in the inventory plots. Such is the case of *Anonidium mannii* Engl. & Diels, *Gilbertiodendron dewevrei*, *and Ceiba pentandra* (L.) Gaertn. in Yaselia and Lilanda, and *Diospyros crassiflora* Hiern and *Pentaclethra macrophylla* in Bagbanye.

It is certain that species with high utility value are often deeply rooted in the cultural traditions of local communities. Their disappearance could not only jeopardize biodiversity but also have significant repercussions on the social and economic well‐being of the populations that depend on them. Moreover, the depletion of these species could result in the loss of traditional knowledge and cultural practices associated with their use, thus threatening the intangible heritage of these communities. Integrating multi‐use plants into agroforestry systems would help preserve these species while providing economic benefits to farmers. This model, effective in Central Africa, Asia, and the Amazon (Pantera Mosquera‐Losada et al. 2021; Onefeli et al. 2019; Udawatta et al. 2019; Dollinger and Jose 2018; Bajigo and Tadesse 2015), could help protect biodiversity, promote forest regeneration, and prevent the irreversible loss of vital species within the RBY landscape.

### 4.3 Use of Species in the Yangambi Landscape

Despite the decline or disappearance of certain valuable species in village forests, and faced with the imperative need to survive, the population is called upon to seek out the products of these useful trees far away in the forest. This situation is exacerbated by the lack of demarcation between village exploitation zones and the conservation areas in the YBR, as observed by Kipute et al. (2021). This has led to frequent conflicts between local communities and YBR managers regarding incursions into the protected area. These tensions are further amplified by the absence of sustainable management policies for these species at the local level.

In addition, there is no management policy for these species at the village level. In the past, the village of Lilanda has benefited from 231 *Pentaclethra macrophylla* seedlings, planted as part of the REDD Isangi project (FFBC 2014). Currently, these trees are difficult to identify due to a lack of maintenance and follow‐up after the project ended. This lack of continuity underscores the importance of long‐term management strategies, especially for species with multiple uses. A comprehensive assessment of the utility of these species by use category, as suggested by Yarnvudhi et al. (2016), is essential for their inclusion in reforestation and conservation policies.

Furthermore, Mapenzi et al. (2023) emphasized the need to combine the use of local and exotic species to meet the livelihood needs of local communities while achieving reforestation goals. In this regard, the experience of the CAMPFIRE program in Zimbabwe, where a significant portion of the revenue was redistributed to local communities, has demonstrated that such an approach can significantly reduce poaching and increase local support for sustainable resource management (Child 1996). Implementing a similar program in peripheral villages around the YBR could offer sustainable economic development opportunities, generate income for local communities, and reduce pressure on YBR resources, thereby contributing to biodiversity conservation while improving local livelihoods.

## 5 Conclusion

The ethnobotanical survey conducted in the villages of Yaselia, Lilanda, and Bagbanye highlighted the importance of species used by local communities in the Yangambi landscape. These surveys were coupled with forest inventories in three habitats in each village: dense forest, secondary forest, and fallow lands, to assess the availability of species with high use value. A total of 51 plant species, including *Entandrophragma cylindricum*, *Petersianthus macrocarpus*, *Ricinodendron heudelotii*, *Scorodophloeus zenkeri*, *Uapaca guineensis*, *Pericopsis elata* , *Gilbertiodendron dewevrei*, *Prioria balsamifera*, *Dacryodes edulis* , and *Chrysophyllum lacourtianum*, were identified for their wide range of uses, high use value, and cultural significance. These species mainly belong to the Fabaceae, Euphorbiaceae, and Meliaceae families. Furthermore, the inventory also showed that primary forests host the highest abundance and biomass of these high‐use species, while fallow lands, closer to villages, are less diverse and are dominated by small‐diameter trees.

Thus, the increasing pressure on natural resources in the Yangambi landscape highlights the need to implement participatory development projects focused on sustainable resource management. Policymakers and conservation practitioners must prioritize the promotion of sustainable land‐use practices that integrate high‐use species into reforestation and agroforestry programs. This can help regenerate and conserve these species while simultaneously supporting local livelihoods. Moreover, efforts should be made to implement community‐based conservation strategies that are rooted in traditional knowledge and local expertise. Local communities should be actively involved in the decision‐making processes, ensuring that management strategies align with their cultural and economic needs. Additionally, future studies should focus on evaluating the regeneration potential of multiple‐use species, while also assessing the socio‐economic impact of participatory management practices. This will provide valuable data to refine and strengthen conservation efforts, making them more resilient and adaptive in the face of ongoing environmental pressures.

## Author Contributions

**Daddy D. Kipute:** conceptualization (lead), data curation (lead), formal analysis (lead), funding acquisition (lead), investigation (lead), methodology (lead), writing – original draft (lead), writing – review and editing (lead). **Alain L. Katayi:** investigation (equal), methodology (equal), writing – review and editing (equal). **Nestor K. Luambua:** formal analysis (equal), methodology (equal), writing – review and editing (equal). **Jean‐Marie Kahindo:** supervision (equal), validation (equal), writing – review and editing (equal). **Salomon Mampeta:** conceptualization (equal), methodology (equal), supervision (equal), validation (equal), writing – review and editing (equal). **Ursil Lelo:** supervision (equal), validation (equal), writing – review and editing (equal). **Daou Véronique Joiris:** conceptualization (equal), methodology (equal), supervision (lead), validation (lead), writing – review and editing (equal). **Jean‐Pierre Mate:** conceptualization (equal), funding acquisition (equal), methodology (equal), supervision (lead), validation (lead), writing – review and editing (lead).

## Conflicts of Interest

The authors declare no conflicts of interest.

## Data Availability

The data collected and analyzed for this paper are available on Dryad: https://datadryad.org/stash/share/INsK3o5PNbeQ6r4_B_29b0qjgZilBUbgoDnWNO_1Olg.
